# Left atrial volume during the early convalescent phase of acute MI is strongly related to expansion of myocardial extracellular matrix during infarct healing and ventricular remodeling

**DOI:** 10.1186/1532-429X-15-S1-O78

**Published:** 2013-01-30

**Authors:** Hoshang Farhad, Siddique Abbasi, Ravi Shah, Bobby Heydari, Tomas G Neilan, Jiazuo H Feng, Michael Jerosch-Herold, Raymond Y Kwong

**Affiliations:** 1 Department of Cardiovascular Medicine, Brigham and Women's Hospital/Harvard Medical School, Boston, MA, USA; 2 Cardiology, Massachusetts General Hospital, Boston, MA, USA

## Background

Changes in left ventricular compliance caused by diffuse fibrosis after MI may result in diastolic dysfunction and left atrial enlargement. We sought to test the hypothesis that left atrial volume is associated with ECV (a marker of diffuse myocardial fibrosis) during the sub-acute phase of infarction and can predict the increase of ECV during the ensuing months of infarct healing. We quantified left atrial volumes serially in patients after ST-elevation myocardial infarction and assessed their relationship with indices of post-MI remodeling including left ventricular dimensions, ECV, and infarct size.

## Methods

Sixty-seven patients underwent gadolinium-enhanced cardiac magnetic resonance (CMR) imaging serially at 2-4 weeks and 6 months after STEMI. Left atrial volumes (LAV) were calculated at the end of ventricular systole (LAVmax), just before atrial contraction (LAVbac), and at the end of ventricular diastole (LAVmin) using the biplane area-length method. Using a segmented, breath-held Look-Locker sequence in 3 short axis slices, T1 measurements were made pre- and post-contrast up to 30 minutes after administration of gadolinium (gadopentetate dimeglumine, 0.15 mmol/kg). Regression of myocardial R1 (defined as 1/T1) against R1 of the blood pool was used to determine the gadolinium partition coefficient, which when multiplied by (1-hematocrit/100), to estimate ECV. Total infarct size was measured using a full-width half maximum (FWHM) methodology and was expressed in total grams and %LGE.

## Results

Patients were predominantly male (83%), with a mean age of 58±11 years, and had a mean LVEF of 53±10% (see Table 1). Mean ECV was 0.35 ± 0.07 at the baseline scan. LAV demonstrated a strong positive correlation with ECV (r=0.48, p=0.002), and this relationship maintained after adjustment to patient age and LVEDV (p=0.01). LAV was also positively correlated to infarct size at baseline (r=0.44, p=0.0071). Progression of LAV demonstrated a strong correlation with the progression of ECV expansion during the 6 months of infarct healing (r=0.47, P=0.004).

**Table 1:** Baseline characteristics

Sex	
Male	83%

Female	17%

Age	58±10

Medication use	

Beta-blocker	94%

ACE inhibitor	84%

Statin	97%

Comorbidities	

Diabetes	21%

Hypertension	60%

Dyslipidemia	66%

Tobacco Use	51%

LVEF	52±10%

RVEF	54±7%

LV mass (grams)	118±36

LVEDV	164±47

LVESV	88±44

All values expressed as means or percentages, as appropriate.

## Conclusions

Large left atrial volume is associated with expansion of the extracellular matrix early after infarction, and its progression reflects alteration of ECV during infarct healing. We postulate that left atrial volume sensitively reflects diastolic ventricular stiffness early and during infarction remodeling.

## Funding

Dr. Kwong receives is supported in part by a research grant from the National Institutes of Health (R01HL091157).

Dr. Jerosch-Herold is supported in part by a research grant from the National Institutes of Health (R01HL090634-01A1).

